# A Case Report: Treatment, follow-up, and literature review of type A insulin resistance syndrome caused by a *De Novo* INSR c.3328G>C mutation

**DOI:** 10.3389/fendo.2026.1840942

**Published:** 2026-05-26

**Authors:** Chunmin Ji, Xiaojun Zheng, Quanxing Wang, Zhongyun Chen

**Affiliations:** Department of Endocrinology, Quanzhou Hospital of Traditional Chinese Medicine, Quanzhou, China

**Keywords:** case report, gene c.3328G > C mutation, literature review, severe insulin resistance, treatment and follow-up, type A insulin resistance syndrome

## Abstract

**Background:**

Type A insulin resistance syndrome (TAIRS) is a rare inherited disorder caused by mutations in the insulin receptor (INSR) gene. It is characterized by severe insulin resistance, hyperandrogenism, and acanthosis nigricans. Its clinical manifestations overlap with those of polycystic ovary syndrome (PCOS), making it prone to misdiagnosis, particularly in adolescent females. Currently, the diagnosis of TAIRS relies on clinical evaluation and genetic confirmation, while long-term management strategies remain limited and challenging.

**Case presentation:**

We report a 12-year-old female proband who presented with severe hyperinsulinemia, hyperandrogenism (hirsutism), acanthosis nigricans, and polycystic ovarian changes despite a non-obese phenotype. Whole-exome sequencing identified a *de novo* heterozygous variant in the INSR gene (NM_000208.4:c.3328G>C, p.Asp1110His). This variant was absent from the ClinVar and gnomAD databases and was predicted to be deleterious by in silico analyses. Family segregation analysis demonstrated that neither parent nor the proband’s brother carried the variant, consistent with a *de novo* event. According to ACMG guidelines, this variant was classified as “likely pathogenic,” providing a molecular basis for the diagnosis of TAIRS.

**Conclusions:**

This case is attributed to a *de novo* p.Asp1110His variant in INSR. The diagnostic process highlights the importance of genetic testing in adolescents presenting with atypical PCOS features, particularly severe hyperinsulinemia and non-obesity. This report expands the known pathogenic variant spectrum of the INSR gene and reviews TAIRS cases reported since 2010. It further emphasizes that long-term management of TAIRS requires individualized, multidisciplinary strategies, including pharmacological and medical nutrition therapy in addition to lifestyle interventions. Type A insulin resistance syndrome (TAIRS) is a rare genetic disorder resulting from mutations in the insulin receptor (INSR) gene and may be inherited in either an autosomal dominant or autosomal recessive manner. The disorder is primarily characterized by severe insulin resistance, accompanied by clinical features such as hyperandrogenism, hirsutism, and acanthosis nigricans. In female patients, polycystic ovary syndrome (PCOS) is a common associated condition. Early diagnosis and intervention are crucial, as they can delay the onset of precocious puberty and reduce the risk of metabolic and endocrine complications, as well as related neoplasms. This article reports a TAIRS proband carrying a novel mutation, with the aim of providing a reference for the clinical diagnosis and management of this rare condition.

## 1 Medical records

### 1.1 General circumstances

The proband was a 12-year-old female born in Quanzhou, Fujian Province, with no history of long-term residence elsewhere. She was delivered at full term with a birth weight of 2.4 kg and a birth length of 50 cm. The mother experienced no complications during pregnancy, and the parents were non-consanguineous. There was no family history of diabetes mellitus or other hereditary disorders. Both parents and the patient’s brother exhibited normal growth and development.

The patient had exhibited excessive body hair since early childhood, primarily involving the limbs, upper lip, and trunk. In September 2023, hyperpigmentation developed in the skin folds of the neck and axillary regions. In July 2024, she was treated with unspecified oral traditional Chinese medicine at another medical institution, after which she experienced a small amount of brown vaginal discharge that resolved spontaneously within 2–3 days. Menarche occurred in October 2024, followed by a prolonged menstrual period lasting more than 20 days, with no subsequent recurrence. Over the past year, the patient’s height increased by approximately 15 cm, accompanied by deepening of the voice, alopecia, and acne on the back. She denied symptoms of xerostomia, polydipsia, rapid weight gain, abnormal tooth development, galactorrhea, or headache.

### 1.2 Physical examination

**Figure 1:**
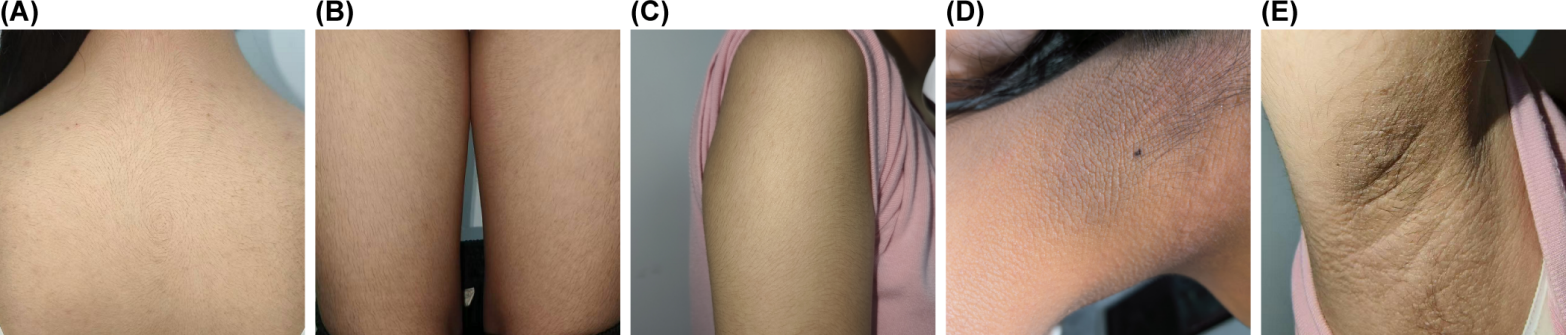
**(A)** upper back. **(B)** thigh; **(C)** upper arm; **(D)** neck acanthosis nigricans sign; **(E)** axillary acanthosis nigricans sign.

On physical examination, the patient’s height was 157 cm and weight was 44 kg, corresponding to a body mass index of 17.85 kg/m² (75th percentile). Marked acanthosis nigricans was observed in the skin folds of the neck, axillae, and bilateral knees. Fine vellus hair was present on the upper lip, while coarse, virilized terminal hair was distributed over the chest, abdomen, back, upper limbs, and thighs. The Modified Ferriman-Gallwey (mFG) score was 16, indicating significant hirsutism. The skin of the posterior trunk showed enlarged pores with scattered acneiform lesions. Breast development was appropriate for age. Pubic hair was dense and distributed in an inverted triangular pattern, accompanied by clitoral hypertrophy (**Figures 1A–E**).

### 1.3 Results of laboratory examination, imaging examination and genetic testing

Cardiac, hepatic, and renal function tests, as well as serum lipid profiles and electrolyte levels, were within normal ranges. A 75-g oral glucose tolerance test indicated impaired glucose tolerance (**Table 1**), accompanied by markedly elevated serum insulin levels, a homeostasis model assessment of insulin resistance (HOMA-IR) index of 30.2, and negative results for glutamic acid decarboxylase antibodies, insulin autoantibodies, and islet cell antibodies. Thyroid function, sex hormone levels, cortisol circadian rhythm, adrenocorticotropic hormone, and steroid hormone profiles were assessed, with the results summarized in **Table 2**.

**Table 1:** OGTT test of the proband.

Variable	0min	30min	60min	120min	180min
Blood glucose mmol/L	4.44	9.93	10.17	9.98	7.81
Insulin mu/L	153.1	>300	>300	>300	>300

**Table 2:** Hormone levels in the proband.

Name of hormone	Test results	Reference range	Hormone levels	Test results	Reference range
Cortisol 08:00	11.82ug/dl	7:00-9:00:4.26-24.85	estradiol	60.3pg/ml	19.5-144.2
Cortisol 16:00	5.28ug/dl	15:00-17:00:2.9-17.3	Follicle stimulating hormone	7.1mIU/ml	2.5-10.2
Cortisol 00:00	0.84ug/dl		Luteinizinhormone horLuteinizing hormone Luteinizing horm	15.4mIU/ml	1.9-12.5
Adrenocorticotropic hormone 08:00	7.5pg/ml	7:00-10:00:7.2-63.4	Prolactin	10.7ng/ml	2.8-29.2
Hypersensitive thyrotropin	1.399mIU/L	2.3-4.2	Total testosterone	2026.6pg/ml	<750
Free T4	11.96pg/ml	8.9-17.6	Androstenedione	2642.3pg/ml	420-1000
FreeT3	4.3pg/ml	2.3-4.2	Dehydroepiandrosterone sulfate	289ng/ml	220-1840
17-hydroxyprogesterone	2265.0pg/ml	<1000	Dehydroepiandrosterone	2568.2pg/ml	<5000
Testosterone	131.8ng/dl	7-27.57			

Imaging Studies Electrocardiography demonstrated sinus rhythm with an anticlockwise axis deviation. Adrenal computed tomography revealed normal bilateral adrenal morphology. Gynecological ultrasonography showed polycystic changes in both ovaries, characterized by more than ten anechoic follicles measuring ≤8 mm in diameter and arranged in a radial pattern on the transverse plane. No significant abnormalities were observed in the uterus or in abdominal organs, including the liver, gallbladder, pancreas, and spleen.

**Figure 2:**
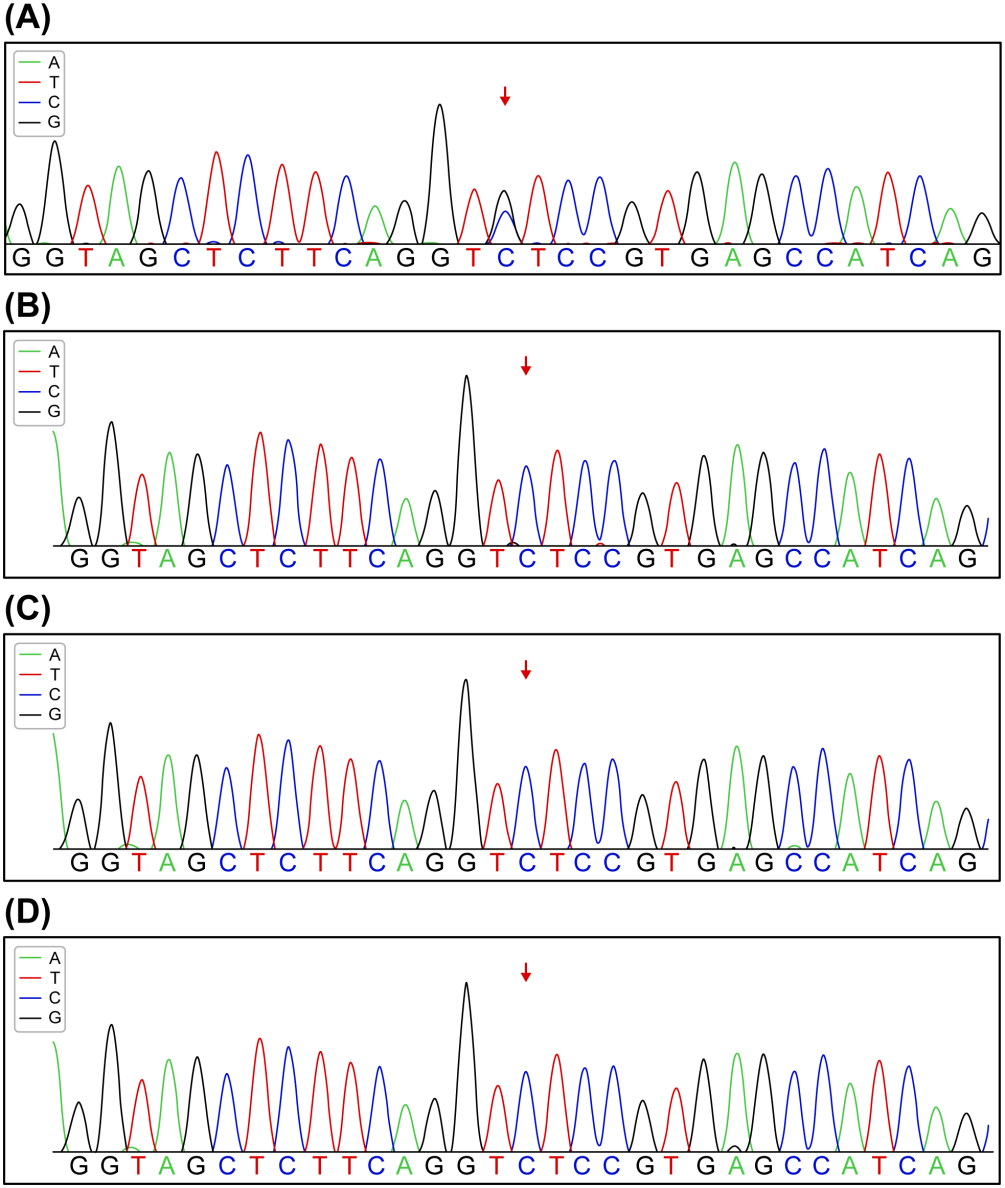
**(A)** Gene verification map of the proband. **(B)** The gene map of the proband’s father. **(C)** The gene map of the proband’s mother. **(D)** The gene map of the proband’s younger brother.

Genetic testing: Whole-exome sequencing combined with multiplex ligation-dependent probe amplification (MLPA) of the CYP21A2 gene identified a heterozygous variant in the INSR gene (NM_000208.4: c.3328G>C, p. Asp1110His). Confirmatory genetic testing demonstrated that the variant was absent in both parents and the proband’s younger brother. MLPA analysis of the CYP21A2 gene revealed no definitive deletions, duplications, or common pathogenic point mutations within the regions covered by the probes (**Figures 2A–D**).

## 2 Treatment plan

1. Dietary intervention: Adjustment of staple foods to increase the intake of resistant starch-rich foods, including steamed potatoes, cooled sweet potatoes, oats, corn, and similar sources.
2. Metformin hydrochloride tablets 0.5g tid were taken orally

## 3 Follow-up results

1. Clinical symptoms: Following treatment, the roughness and hyperpigmentation of the skin folds in the neck and axillary regions improved markedly, with a significant reduction in hyperpigmentation observed over both knees. Menstruation resumed during the second month of therapy, with normal menstrual volume and cycle duration.

2. Assessment of glucose metabolism, islet cell function and testosterone level **Table 3**.

**Table 3:** Metabolic and hormonal parameters of the proband after treatment.

Variable	0min	30min	60min	120min	180min
Blood glucose mmol/L	4.16	9.12	10.17	8.58	7.85
Insulin mu/L	60.80	124.00	>300	>300	>300
Testosterone ng/dl	16.60(7-27.57)				

3. Follow-up conclusions

Short-term treatment with metformin hydrochloride combined with dietary modification in patients with TAIRS can reduce serum insulin and total testosterone levels and ameliorate associated endocrine abnormalities; Furthermore, no adverse effects, including gastrointestinal discomfort, impaired liver function, or hypoglycemia, were observed during the follow-up period. however, the long-term therapeutic efficacy requires further follow-up and evaluation.

## 4 Discussion

TAIRS is a rare genetic disorder that may be inherited in either an autosomal dominant or autosomal recessive manner. It is caused by mutations in the INSR gene and is clinically characterized by severe insulin resistance, hyperandrogenism, and acanthosis nigricans. The core pathophysiological feature of TAIRS is profound insulin resistance accompanied by compensatory hyperinsulinemia, resulting from impaired INSR function. It is characterized by varying degrees of acanthosis nigricans, hyperandrogenism (including hirsutism, acne, and oligomenorrhea), and impaired glucose metabolism. Affected individuals typically exhibit a normal or lean body habitus and generally do not present with the dyslipidemia or hepatic steatosis commonly associated with metabolic syndrome (1). The clinical features observed in this patient, including severe hyperinsulinemia, hyperandrogenism, and acanthosis nigricans, are consistent with the typical manifestations of TAIRS reported in the literature. However, the presence of clitoral hypertrophy is a notable finding, suggesting a broader endocrine involvement to be considered in the differential diagnosis and highlighting the phenotypic specificity of this case. Even among individuals carrying identical mutations, the clinical presentation of TAIRS shows marked heterogeneity. Disease severity is strongly influenced by modifying factors, including genetic background, environmental influences, and sex (2–6).

TAIRS is mainly caused by mutations in the insulin receptor gene (INSR). The mutation spectrum of INSR is highly heterogeneous. To date, hundreds of INSR variants have been identified, with more than one hundred classified as pathogenic. These include missense, nonsense, deletion, insertion, and splice-site variants (2, 7, 8). In terms of inheritance, TAIRS is primarily autosomal dominant, although *de novo* mutations have also been reported (1, 9). The patient described in this study also harbors a *de novo* variant. From the perspective of variant pathogenicity, approximately 60% of pathogenic variants are located in the exonic regions encoding the tyrosine kinase (TK) domain of the β-subunit. Variants in this domain predominantly exert a dominant-negative effect, disrupting normal insulin signaling and representing the principal molecular mechanism underlying the TAIRS phenotype (3–5, 9–11). Therefore, in clinically suspected cases, such as non-obese adolescents presenting with severe insulin resistance, genetic testing of INSR, particularly through whole-exome sequencing, is essential to establish a definitive molecular diagnosis and facilitate precision medicine.

Currently, the long-term management of TAIRS remains challenging due to the absence of standardized treatment protocols. Management is therefore guided by an individualized strategy that integrates pharmacological therapy and complication control, with lifestyle modification as the foundational component (1). Lifestyle intervention, particularly medical nutrition therapy, constitutes the cornerstone of management in all patients (9). Careful dietary regulation and sustained behavioral modification are essential for improving metabolic control and reducing insulin demand. With respect to pharmacotherapy, metformin is commonly employed as a first-line agent to enhance insulin sensitivity. However, therapeutic response varies among patients, and clinical efficacy may be limited in cases with severe receptor-level defects. Thiazolidinediones have shown benefit in some cases, although results remain inconsistent (10, 11). In recent years, sodium-glucose cotransporter 2 (SGLT2) inhibitors, which act independently of insulin signaling, have demonstrated potential to improve glycemic control and reduce insulin requirements in several case reports and are considered promising therapeutic options (12, 13). Hyperandrogenism may be managed with anti-androgen agents (e.g., spironolactone, flutamide) or combined oral contraceptives containing cyproterone acetate (9). When oral hypoglycemic agents are ineffective, high-dose insulin therapy is often required; however, patients frequently exhibit severe insulin resistance. Management during pregnancy is particularly challenging, often necessitating substantial increases in insulin dosage. The combination of metformin and insulin may help reduce insulin requirements and improve glycemic control, although its use should be carefully considered under close monitoring (14). Overall, the management of TAIRS requires a multidisciplinary approach and should account for the variability in treatment response driven by the heterogeneity of both clinical presentation and genetic background.

The patient is an adolescent female with a non-obese phenotype and a history of normal growth and development. Her clinical presentation was characterized by hirsutism, acanthosis nigricans, marked hyperinsulinemia, severe insulin resistance, hyperandrogenism, and polycystic ovarian morphology. Before a diagnosis of type A insulin resistance syndrome is established, other conditions with similar clinical manifestations should be systematically excluded. The patient showed no evidence of localized or generalized lipodystrophy, no history of prolonged glucocorticoid exposure, and no signs of severe malnutrition. Clinical evaluation, biochemical testing, thyroid function assessment, and serum cortisol levels were all within normal ranges, thereby making lipodystrophy and endocrine disorders such as Cushing’s syndrome and thyroid dysfunction unlikely. In addition, the absence of significant hyperglycemia or hypoglycemia, together with no clinical features suggestive of autoimmune disease (e.g., xerostomia, xerophthalmia, facial rash, or arthralgia/arthritis), rendered type B insulin resistance syndrome highly improbable based on the overall phenotype. Given the clinical profile, the primary conditions requiring careful differentiation were congenital adrenal hyperplasia (CAH) and PCOS. Physical examination revealed clitoromegaly, accompanied by mildly elevated 17-hydroxyprogesterone levels. To exclude 21-hydroxylase deficiency, whole-exome sequencing was supplemented with multiplex ligation-dependent probe amplification (MLPA) analysis of the CYP21A2 gene. The MLPA results demonstrated no definitive deletions, duplications, or common pathogenic point mutations within the probe-covered regions, effectively excluding classic CAH due to 21-hydroxylase deficiency. Regarding PCOS, although the patient exhibited hyperandrogenism and polycystic ovarian morphology, she had not yet established regular menstrual cycles, and these findings may reflect physiological characteristics of adolescence. Moreover, the severity of hyperinsulinemia was far beyond that typically observed in adolescent PCOS. Collectively, the available evidence was insufficient to support a diagnosis of PCOS at this stage. Based on the overall clinical and biochemical profile, the diagnostic focus shifted toward TAIRS. Whole-exome sequencing identified a heterozygous variant in the INSR gene (NM_000208.4:c.3328G>C, p.Asp1110His) in the proband. This variant is absent from both the ClinVar and gnomAD databases. According to the American College of Medical Genetics and Genomics (ACMG) guidelines, the variant was classified using the following criteria: PP3_Strong (multiple in silico tools predicting deleteriousness, with a REVEL score ≥ 0.932), PM2_Supporting (absence from population databases such as gnomAD), and PM6_Supporting (*de novo* occurrence). Familial segregation analysis confirmed that neither parent nor the younger brother carried the variant, supporting a *de novo* origin. Based on the combined evidence, the variant was classified as likely pathogenic. This molecular finding provided a definitive basis for the diagnosis of TAIRS and confirmed the initial clinical suspicion. Overall, this case highlights the importance of a structured differential diagnostic approach and the critical role of genetic testing in establishing an etiological diagnosis in adolescents presenting with atypical features of PCOS, particularly in the presence of severe insulin resistance without obesity.

Summarizing treatment experiences reported in previous case studies and considering the characteristics of this patient, metformin was selected based on several considerations. First, previous studies have demonstrated its favorable efficacy and safety profile in adolescents with severe hyperandrogenism (15). Second, given the increased long-term risks of osteoporosis, tumor development, and accelerated aging in patients with TAIRS, metformin may offer additional benefits beyond improving insulin sensitivity. These include activation of the AMP-activated protein kinase (AMPK) pathway, improved glycemic control with reduced accumulation of advanced glycation end products, and modulation of the insulin-like growth factor-1 (IGF-1) signaling pathway, which may confer potential bone-protective and anti-aging effects (16, 17).

After three months of treatment with metformin combined with dietary intervention, primarily aimed at increasing the intake of resistant starch, the patient’s metabolic status improved significantly. This was evidenced by reductions in fasting insulin levels and insulin resistance, normalization of testosterone levels, and restoration of regular menstruation. This overall improvement is attributable to a comprehensive, multimodal intervention. Although metformin exerted a significant pharmacological effect, the individualized dietary regimen implemented for the patient, characterized by an increased intake of resistant starch, likely provided an important synergistic contribution to the observed clinical response. As a fermentable dietary fiber, potato-derived resistant starch is metabolized by the gut microbiota to produce short-chain fatty acids, such as butyrate. These metabolites have been shown to improve insulin resistance by activating the AMPK/PI3K-AKT signaling pathway and enhancing glucose transport via GLUT2 and GLUT4 transporters (18). Moreover, animal studies have demonstrated that resistant starch from various sources, including potato and banana, can effectively improve glucose tolerance and insulin sensitivity in experimental models (19, 20). Therefore, in addition to conventional pharmacological treatment, individualized dietary supplementation with resistant starch may represent a safe and potentially effective long-term adjunctive management strategy for patients with TAIRS.

In conclusion, TAIRS is a hereditary disorder that requires lifelong management. Its diagnosis depends on the recognition of characteristic clinical features and must be confirmed through a systematic differential diagnostic approach combined with genetic testing. This case report describes the diagnostic process in a patient with TAIRS caused by a *de novo* INSR variant (p.Asp1110His). The findings indicate that early intervention with metformin, in combination with medical nutrition therapy emphasizing increased intake of resistant starch, may effectively improve insulin resistance and associated endocrine–metabolic abnormalities in such patients. These observations suggest that an integrated management strategy, combining pharmacotherapy with targeted dietary interventions, may represent a promising direction for future research in the treatment of hereditary insulin resistance syndromes.

## Data Availability

The original contributions presented in the study are included in the article. Further inquiries can be directed to the corresponding author.
